# Supplementary material for: Hydrogenated Cs2AgBiBr6 for significantly improved efficiency of lead-free inorganic double perovskite solar cell
Source: Nat Commun. 2022 Jun 13;13:3397. doi: 10.1038/s41467-022-31016-w (PMC9192601; doi:10.1038/s41467-022-31016-w)
Supplement: Supplementary file 1 — Supplementary Information [file 41467_2022_31016_MOESM1_ESM.docx]

**Supplementary Information**

Hydrogenated Cs_2_AgBiBr_6_ for Significantly Improved Efficiency of Lead-Free Inorganic Double Perovskite Solar Cell

Zeyu Zhang^1^, Qingde Sun^2^, Yue Lu^1*^, Feng Lu^3^, Xulin Mu^1^, Su-Huai Wei^2*^, Manling Sui^1*^

^1^Beijing Key Laboratory of Microstructure and Properties of Solids, Faculty of Materials and Manufacturing, Beijing University of Technology, Beijing, 100124, China

^2^Beijing Computational Science Research Center, Beijing 100193, China

^3^Department of Electronic Science and Engineering, and Tianjin Key Laboratory of Photo-Electronic Thin Film Device and Technology, Nankai University, Tianjin 300071, China

*Corresponding author: [luyue@bjut.edu.cn](mailto:luyue@bjut.edu.cn), [suhuaiwei@csrc.ac.cn](mailto:suhuaiwei@csrc.ac.cn), [mlsui@bjut.edu.cn](mailto:mlsui@bjut.edu.cn)


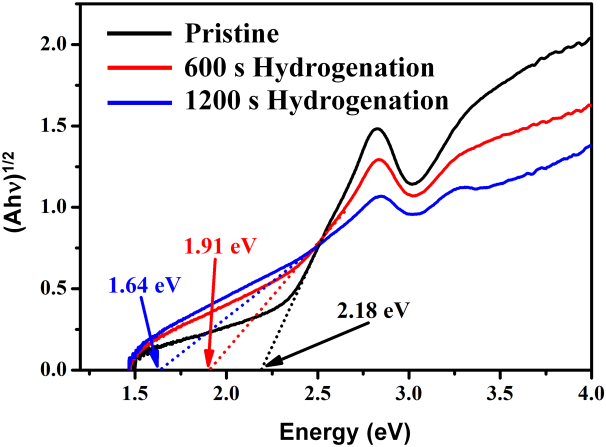


**Supplementary Figure 1.** Tauc plots of the Cs_2_AgBiBr_6_ perovskite films with different hydrogenation time, which is calculated through the indirect bandgap method.


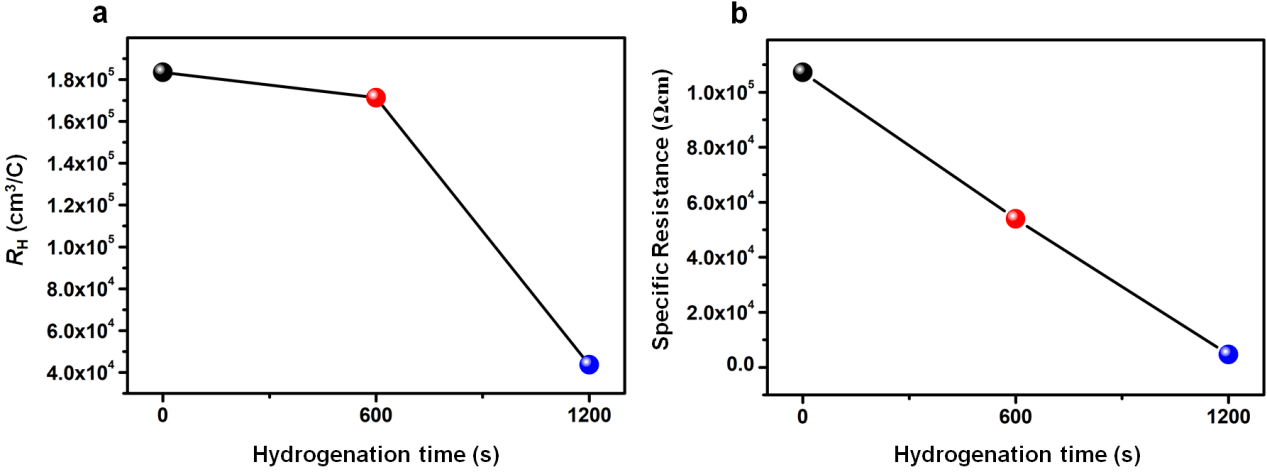


**Supplementary Figure 2. a** The hall coefficient (*R*_H_) and **b** specific resistance of the Cs_2_AgBiBr_6_ perovskite films with different hydrogenation time, which were gained from the hall effect measurement. The carrier mobility was calculated by the equation: Carrier mobility=*R*_H_/Specific Resistance.


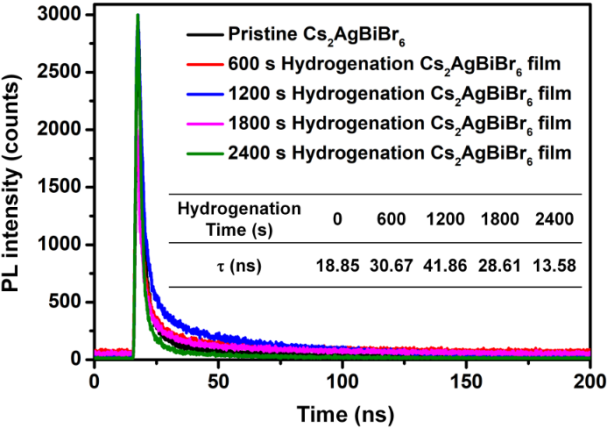


**Supplementary Figure 3.** Time-resolved photoluminescence (TRPL) of the Cs_2_AgBiBr_6_ perovskite films with different hydrogenation time. After 600 s and 1,200 s hydrogenation treatment, the carrier lifetime of Cs_2_AgBiBr_6_ perovskite film increased from 18.85 ns to 30.67 ns and further to 41.86 ns, indicating a proper hydrogenation treatment could improve the transmittability of charge carriers. However, with the further increase of hydrogenation time, the carrier lifetime exhibited a decreasing tendency, which may be caused by the appearance of CsBr impure phase as shown in Fig. 4.


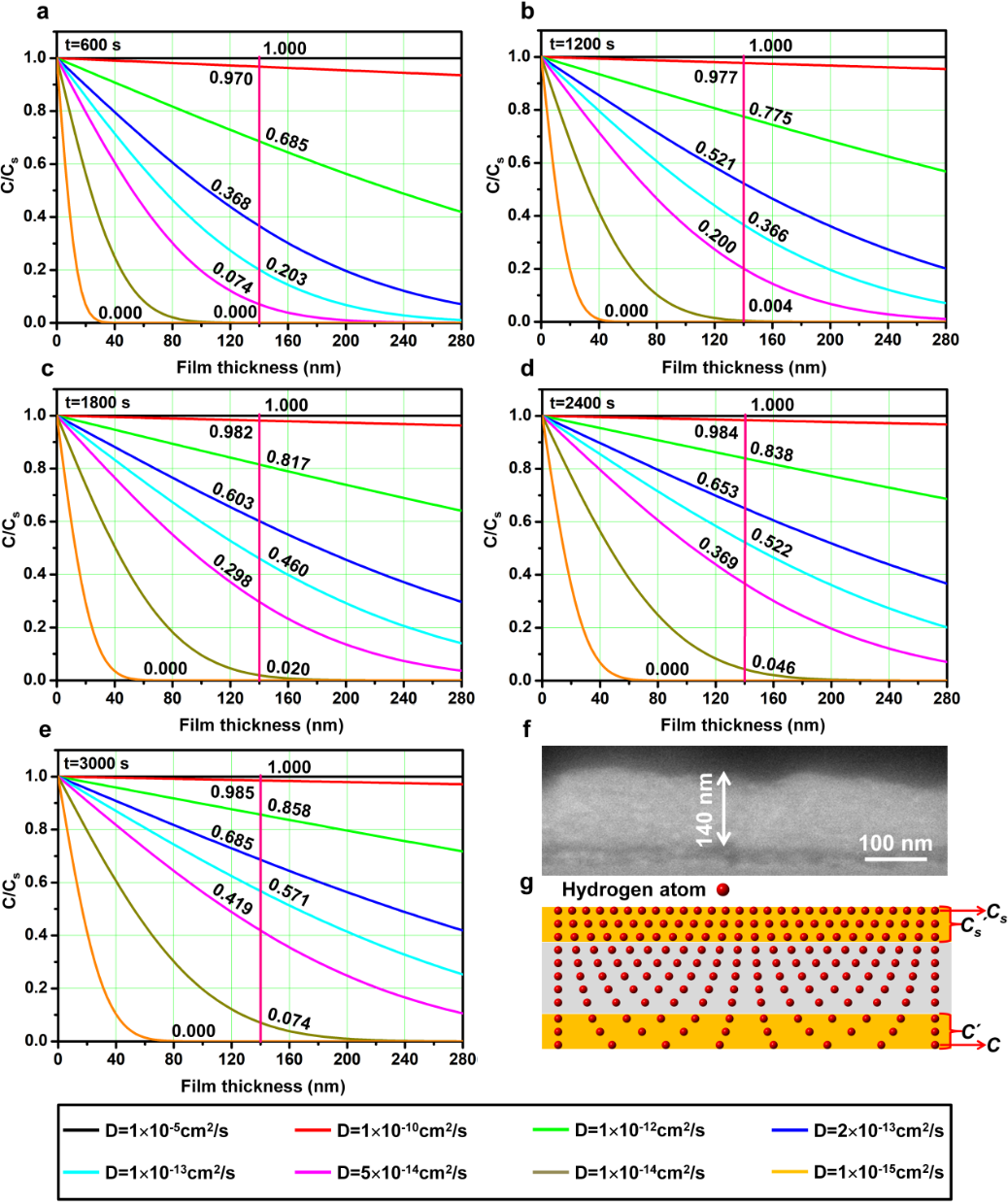


**Supplementary Figure 4. a-e** The calculated depth-dependent $\frac{C}{C_{s}}$ values based on the selected diffusion coefficients from 1×10^-15^ cm^2^/s to 1×10^-5^ cm^2^/s at the different hydrogenation times (600 s, 1,200 s, 1,800 s, 2,400 s and 3,000 s), respectively. **f** Cross-sectional scanning electron microscope (SEM) image confirms that the Cs_2_AgBiBr_6_ film is with a thickness of about 140 nm. **g** Schematic diagram presents the depth-dependent H^*^ concentration distribution in the hydrogenated Cs_2_AgBiBr_6_ film. Due to the thickness of Cs_2_AgBiBr_6_ film is about 140 nm, then the $\frac{C}{C_{s}}$ values at 140 nm for different coefficients were indicated in figures and then summarized in Supplementary Table. 3. Hydrogen atom is represented by the red dot. *C_s_* and *C* represent the hydrogen concentration on the top and bottom surface of hydrogenated Cs_2_AgBiBr_6_ film, respectively. Assuming the blackness values of the front and back side images (in Fig. 2a) are respectively proportional to the average H^*^ concentration near the top and bottom surface of hydrogenated Cs_2_AgBiBr_6_ film, as shown by *C_s_ˊ* and *Cˊ*, then the blackness ratio of the back and front side images can be used as the reference ratio of $\frac{C'}{C_{s}'}$ for judging the rationality of the calculated $\frac{C}{C_{s}}$values.


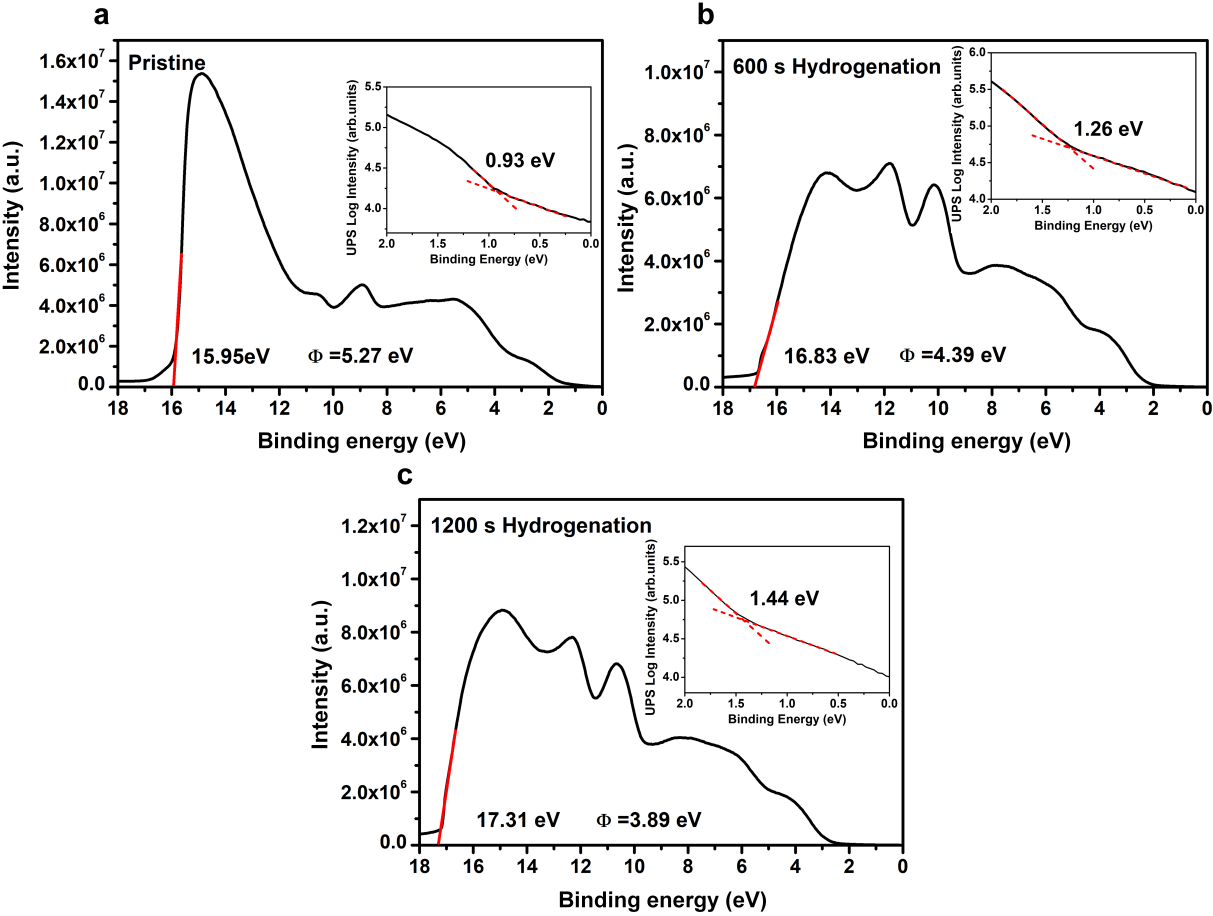


**Supplementary Figure 5. Work function** ***φ*** **of the Cs_2_AgBiBr_6_ perovskite films with different hydrogenated time. a** 0 s, **b** 600 s and **c** 1,200 s (insert shows the energy difference *∆E* between *E_F_* and *E_VBM_* from the valence band spectra). The energy level of VBM value could be calculated through: *E_VBM_* =*φ*+*∆E*. Then according to the bandgap value of the hydrogenated Cs_2_AgBiBr_6_ perovskite films (Fig. 1), the energy level of CBM value could also be determined.


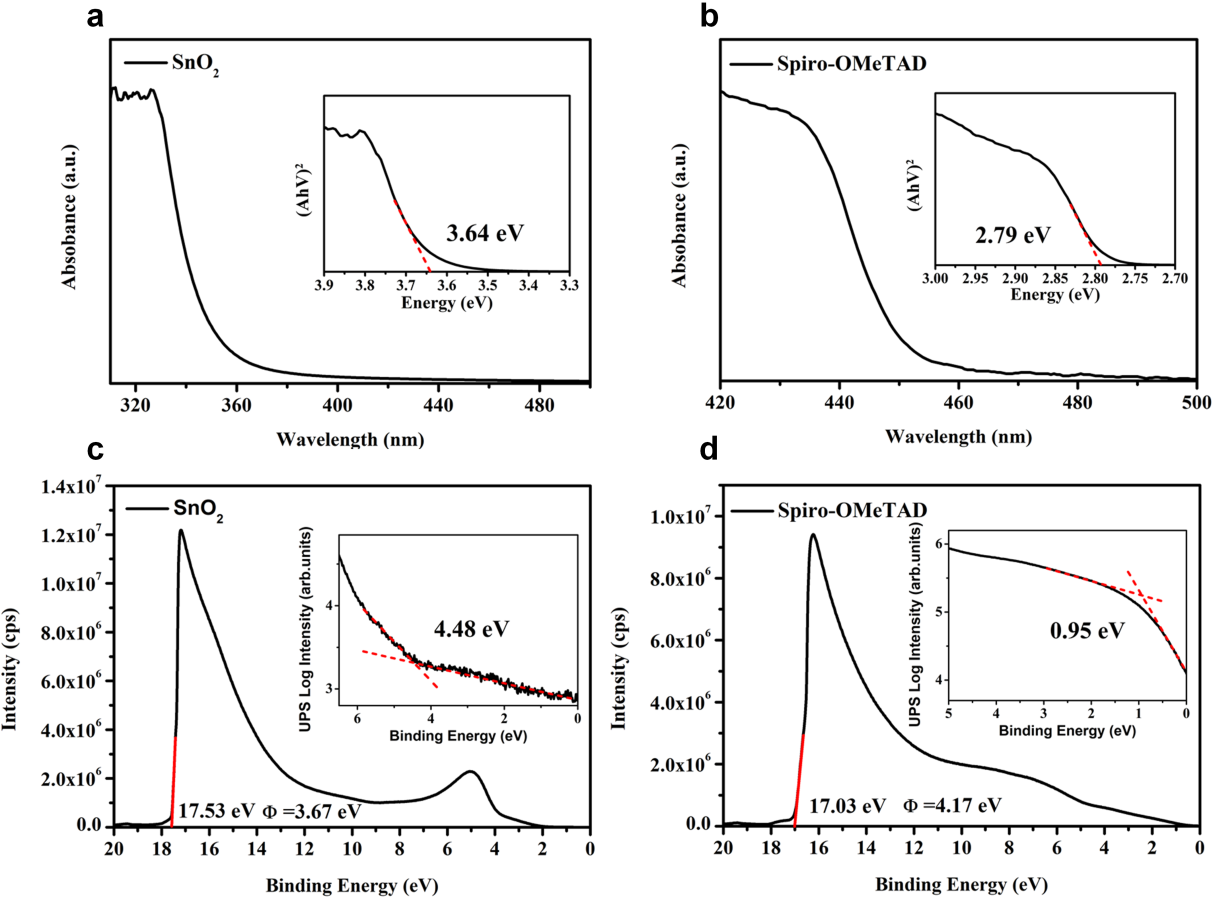


**Supplementary Figure 6.** **a, b** Ultraviolet-visible absorption spectra (UV-vis) of SnO_2_ and Spiro-OMeTAD layers, inserts show the tauc plots of their band gap. **c, d** Work function *φ* of SnO_2_ and Spiro-OMeTAD films, inserts show the energy difference *∆E* between *E_F_* and *E_VBM_*. In order to figure out the applicability of SnO_2_ and Spiro-OMeTAD as ETL and HTL on fabricating the hydrogenated Cs_2_AgBiBr_6_ PSCs, their specific energy levels were determined in Supplementary Fig. 6 and Fig. 2f. Firstly, the bandgap of SnO_2_ layer was measured to be 3.64 eV and *E_VBM_* to be -8.15 eV, so the *E_CBM_* (-4.51 eV) of SnO_2_ matches well with the energy level of hydrogenated Cs_2_AgBiBr_6_ to be a good candidate of ETL. Meanwhile, the bandgap of Spiro-OMeTAD is 2.79 eV and *E_VBM_* is -5.12 eV, that is applicable as the HTL for fabricating hydrogenated Cs_2_AgBiBr_6_ PSC devices (Fig. 2f). So here, PSCs with differently hydrogenated Cs_2_AgBiBr_6_ (0 s, 600 s and 1,200 s) films were designed as ITO/SnO_2_/perovskite/spiro-OMeTAD/Au layered structures to check their photoelectric conversion efficiency (PCE).


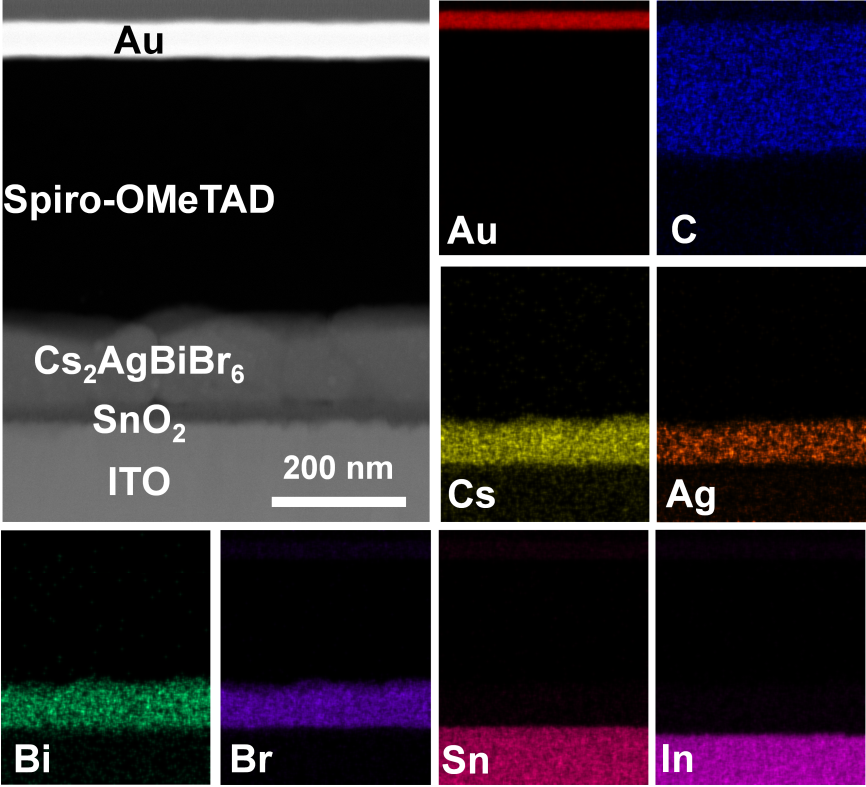


**Supplementary Figure 7.** High-angle annular dark field (HAADF) image and corresponding EDS mapping of the cross-sectional sample of hydrogenated Cs_2_AgBiBr_6_ perovskite solar cell.


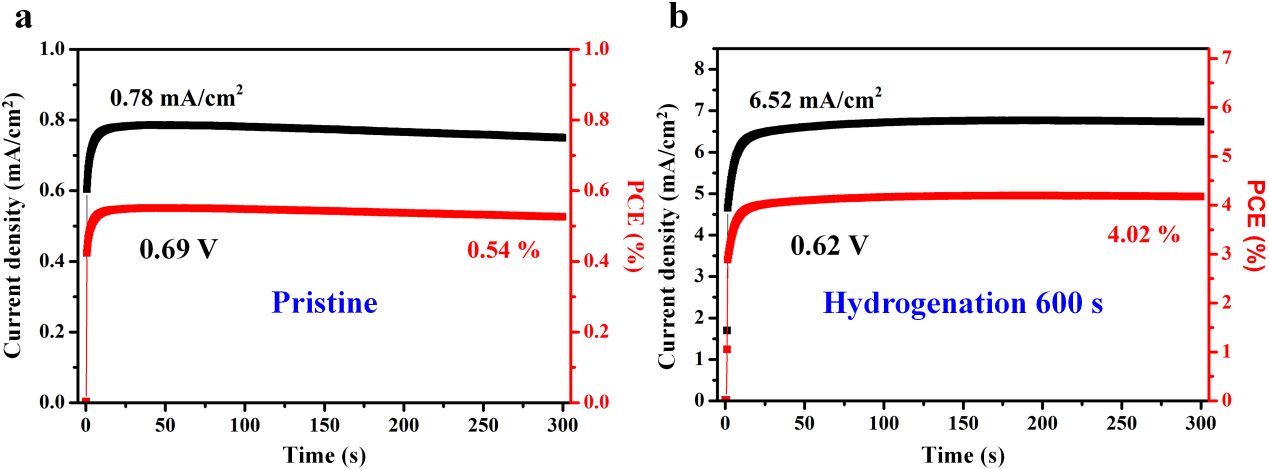


**Supplementary Figure 8.** Steady-state photocurrents of pristine **a** and 600 s **b** hydrogenated Cs_2_AgBiBr_6_ PSCs at bias voltages of 0.69 V and 0.62 V near the maximum power output. Both the pristine and 600 s hydrogenated Cs_2_AgBiBr_6_ PSC devices exhibit a rapid response after light soaking, resulting in stable PCE (about 0.54% for pristine and 4.02% for 600 s hydrogenated sample) and current density (0.78 mA/cm^2^ for pristine and 6.52 mA/cm^2^ for 600 s hydrogenated sample) during the continuous light illumination.


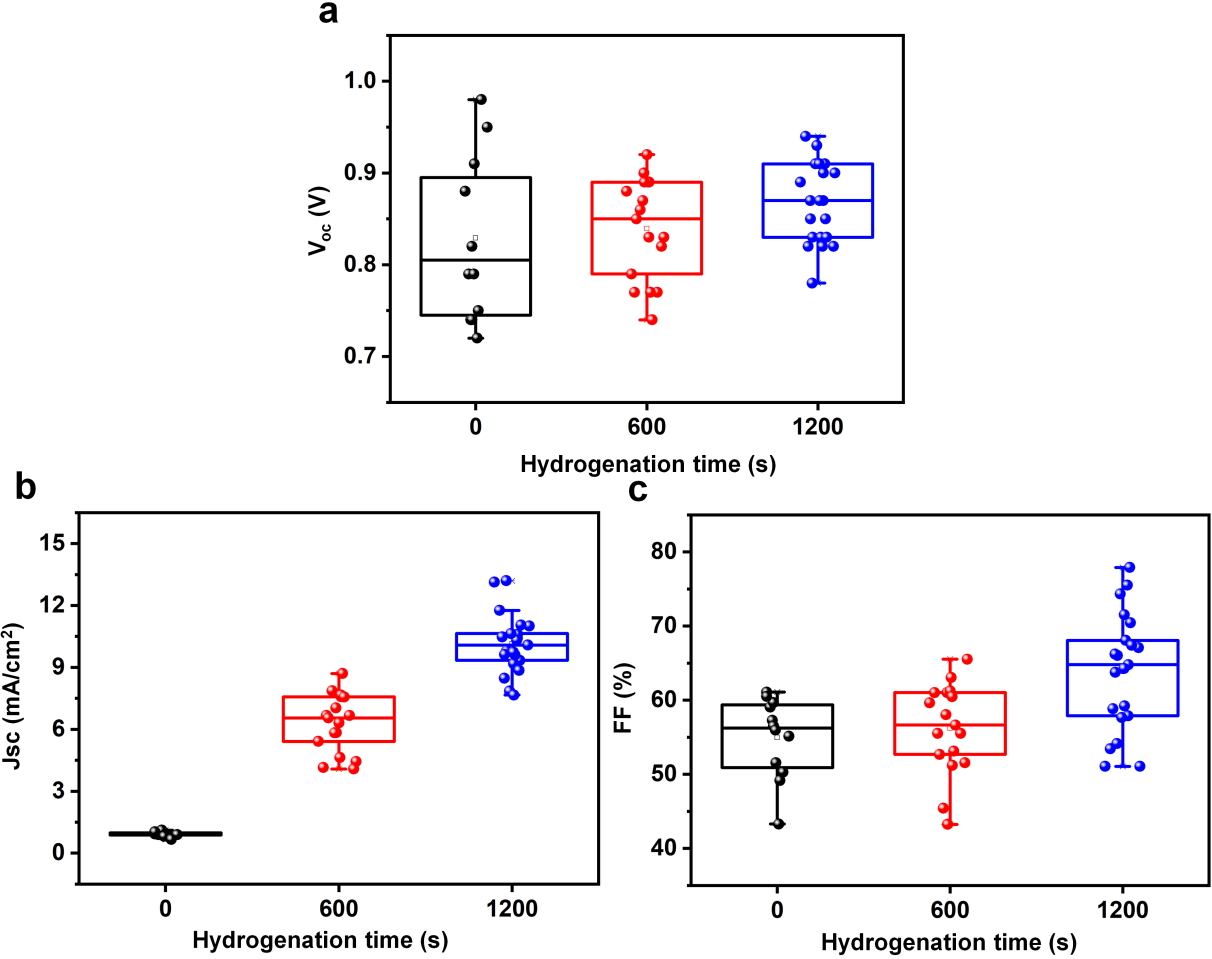


**Supplementary Figure 9.** **a** The average open-circuit voltage (*V_oc_*) distribution of Cs_2_AgBiBr_6_ PSCs with different hydrogenation time (0 s, 600 s and 1,200 s). **b** The average short-circuit current density (*J_sc_*) distribution of Cs_2_AgBiBr_6_ PSCs with 0 s, 600 s and 1,200 s hydrogenation treatment. **c** The average fill factor (FF) of Cs_2_AgBiBr_6_ PSCs with different hydrogenation time (0 s, 600 s and 1,200 s). The middle line, upper/lower box limits and upper/lower whiskers in the box plot indicate the median, 25th/75th quartiles, and maximum/minimum, respectively. The average open-circuit voltage (*V_oc_*) increases from 0.83 V to 0.84 V and then to 0.87 V, the average short-circuit current density (*J_sc_*) increases from 0.92 mA cm^-2^ to 6.33 mA cm^-2^ and then to 10.26 mA cm^-2^ and the FF increases from 54.95% to 56.22% and then to 63.03%, respectively, during the first 1,200 s hydrogenation treatment. All the statistical analysis (at least 50 devices) exhibited the best PSC performance was present on the 1,200 s hydrogenated Cs_2_AgBiBr_6_ PSC.


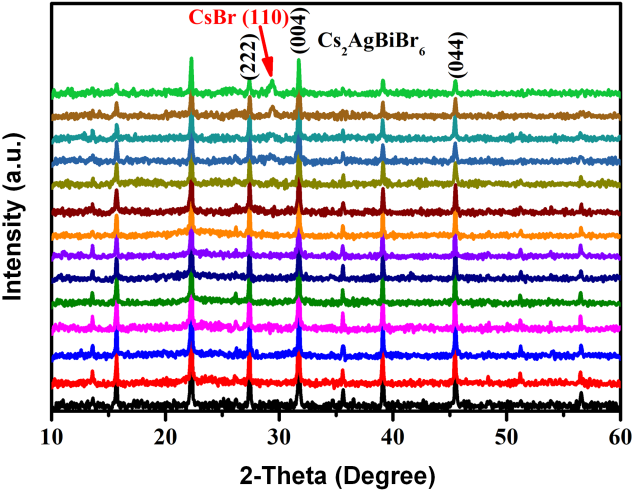


**Supplementary Figure 10.** XRD spectrum of Cs_2_AgBiBr_6_ perovskite films with different hydrogenation time from 0 s to 2,400 s. Red arrow indicates the CsBr (cubic phase with the PDF number of #05-0588) (110) XRD peak (located at 29.365° with the relative intensity of 100%), and the black font of (222), (004) and (044) present the XRD peaks of Cs_2_AgBiBr_6_. It should point out that the BiBr (monoclinic phase, with the PDF number of #34-1171) ($\bar{\text{3}}\text{12}$) XRD peak (located at 29.257° with a relative intensity of 100%) may coexist and overlap with the peak of CsBr (110). Therefore, BiBr may be another decomposition production of Cs_2_AgBiBr_6_ under long-term hydrogenation treatment. According to decomposition products above, the decomposition pathway of Cs_2_AgBiBr_6_ under long-term hydrogenation treatment could be possibly written as: $\text{Cs}_{\text{2}}\text{AgBi}\text{Br}_{\text{6}}\text{+2H}\text{→}\text{2CsBr+2HBr+BiBr+AgBr}$. However, due to the metastable property of AgBr and the weak XRD peak intensity contribution, the existence of AgBr (cubic, PDF number of #06-0438) during the decomposition of Cs_2_AgBiBr_6_ is hard to be identified in this spectrum.


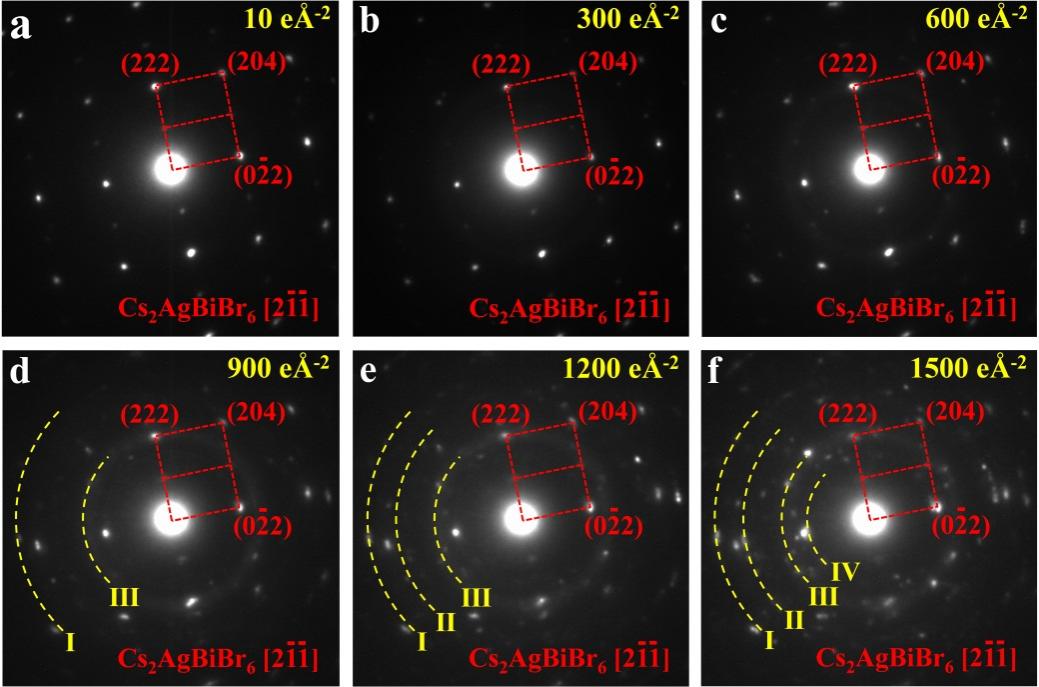


**Supplementary Figure 11.** *In situ* analyses on the evolution of the selected area electron diffraction (SAED) patterns of Cs_2_AgBiBr_6_ under continuous electron beam including **a** 10 e^-^/Å^2^, **b** 300 e^-^/Å^2^, **c** 600 e^-^/Å^2^, **d** 900 e^-^/Å^2^, **e** 1200 e^-^/Å^2^ and **f** 1500 e^-^/Å^2^. Under an electron beam illumination below 300 e^-^/Å^2^, the cube structure of Cs_2_AgBiBr_6_ processes almost no change. However, as increasing the electron dose above 600 e^-^/Å^2^, residual diffraction spot as well as the amorphous diffraction ring become brighter. As elongating the electron dose up to 900 e^-^/Å^2^, the initial diffraction spots of Cs_2_AgBiBr_6_ along [2$\bar{\text{1}}\bar{\text{1}}$] zone axis (marked with red rectangles) became dim, meanwhile new diffraction rings (marked with yellow arcs) I of CsAgBr_2_ (133), AgBr (222) and Cs_3_Bi_2_Br_9_ (042) and III of CsAgBr_2_ (002) and Cs_3_Bi_2_Br_9_ (022) appear gradually. Subsequently, the polycrystal diffraction rings II at 1,200 e^-^/Å^2^ of CsAgBr_2_ (152), AgBr (220) and Cs_3_Bi_2_Br_9_ (220) and IV at 1,500 e^-^/Å^2^ of BiBr_3_ (021) generated. All these results indicate the degradation pathway of Cs_2_AgBiBr_6_ under electron beam irradiation as: (1) 2Cs_2_AgBiBr_6_→CsAgBr_2_+AgBr+Cs_3_Bi_2_Br_9_ and (2) Cs_2_AgBiBr_6_→2CsBr+AgBr+BiBr_3_, which were consistent with others’ reports^1-2^. Therefore, to avoid the beam damage on the TEM characterization of Cs_2_AgBiBr_6_ samples, total electron dose should be controlled below 300 e^-^/Å^2^.


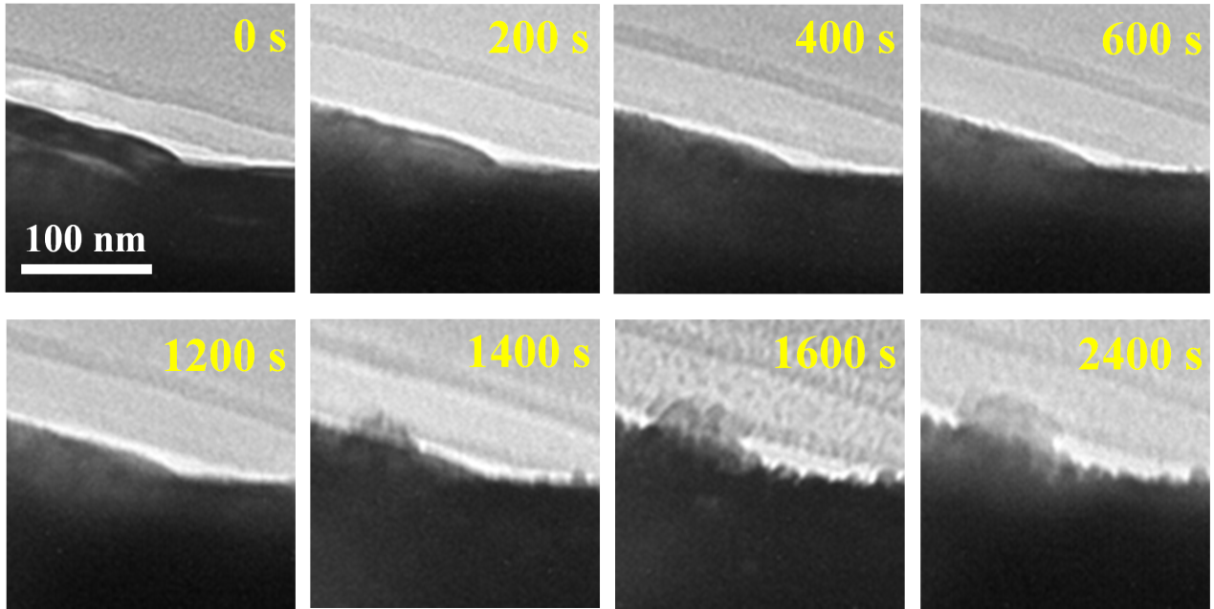


**Supplementary Figure 12.** Quasi *in situ* TEM observation on the morphology evolution of the hydrogenation process of Cs_2_AgBiBr_6_ (corresponding to the SAED patterns in Fig. 4).


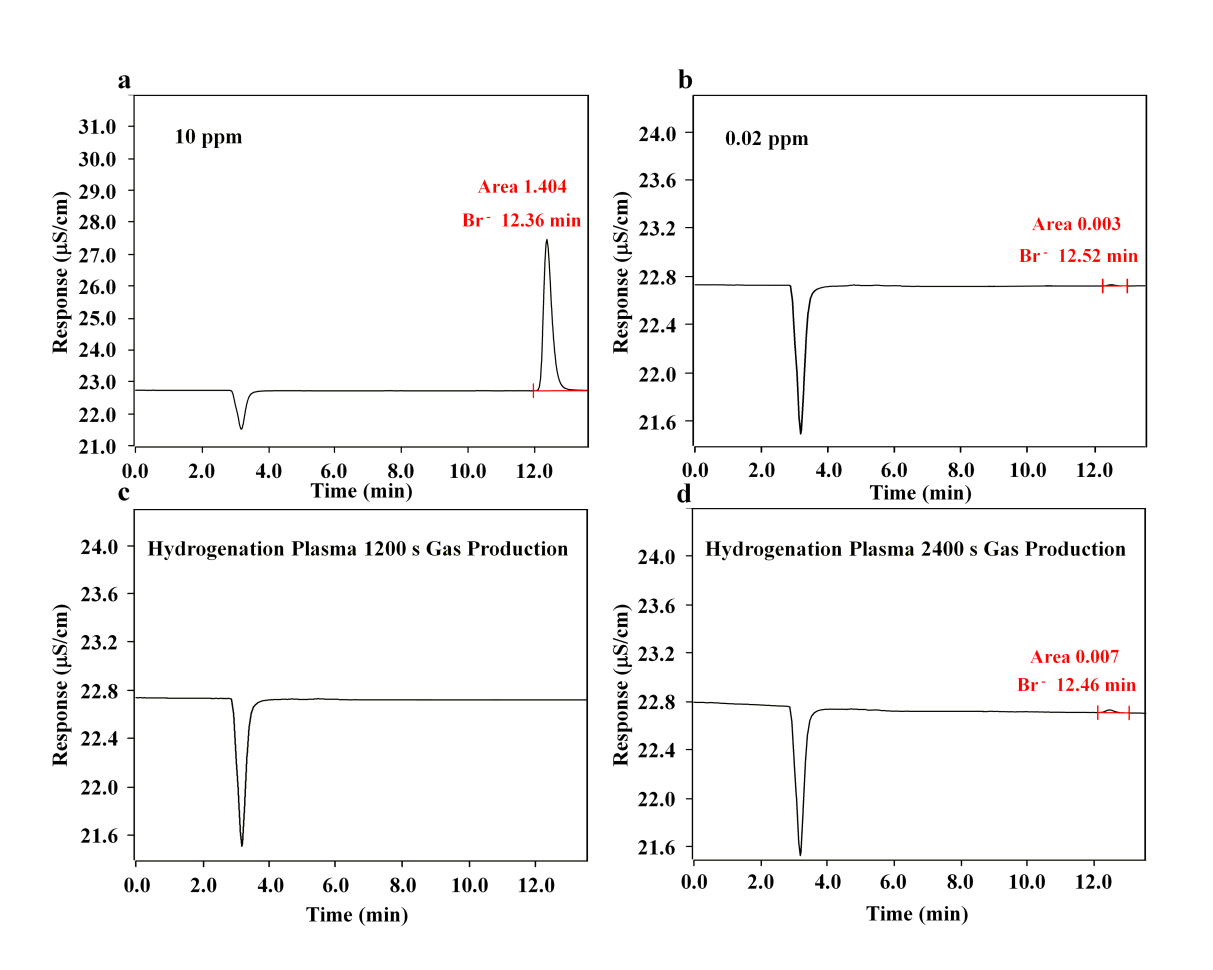


**Supplementary Figure 13.** **a, b** The ion chromatography spectrums of HBr in water solution with concentration of 10 and 0.01 ppm. **c, d** After hydrogenating the Cs_2_AgBiBr_6_ film for 1,200 s and 2,400 s, the gas productions in plasma chamber were collected and proceeded the ion chromatography spectrum detection. Here, the Br^-^ ion deriving from HBr is located at the spectrum position of 12.36-12.52 min. The negative peak at about 3 min indicates the peak of water solution.


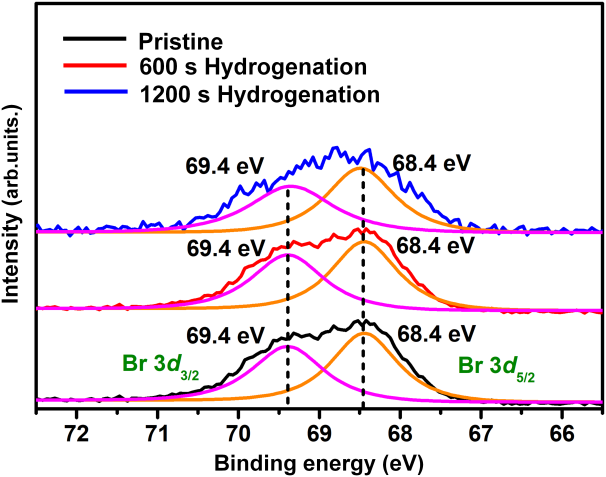


**Supplementary Figure 14.** X-ray photoelectron spectroscopy (XPS) spectra of Br 3*d* in Cs_2_AgBiBr_6_ films with different hydrogenation time (0 s, 600 s and 1,200 s). The binding energy position of Br 3*d* in Cs_2_AgBiBr_6_ films almost unchanged after 600 s and 1,200 s hydrogenation treatment, which means this may be induced by the coexist of H_1_(in), H_2_(in) and H_3_(in) structures in hydrogenated Cs_2_AgBiBr_6_.


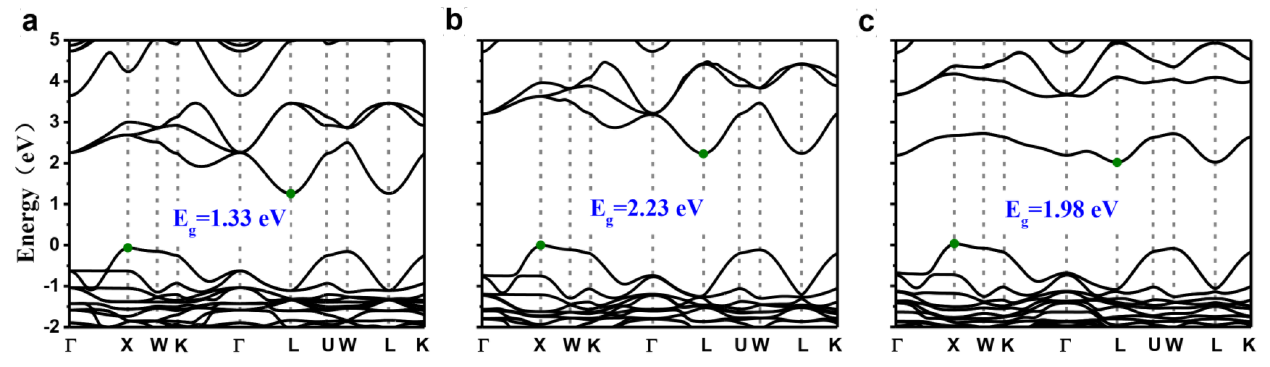


**Supplementary Figure 15. a** PBE, **b** HSE and **c** HSE+SOC band structures of host Cs_2_AgBiBr_6_ in its primitive cell with the bandgaps of 1.33 eV, 2.23 eV, and 1.98 eV, respectively. As we know, the DFT based on GGA calculations could underestimate the bandgaps of crystalline materials. Here, different kinds of models based on HSE and PBE were used to simulate the band structure of host Cs_2_AgBiBr_6_. When compared with the experimental bandgap (2.18 eV) of host Cs_2_AgBiBr_6_ (Fig. 1d), it could find that HSE function (2.23 eV) provides more accurate bandgap than PBE function (1.33 eV). Thus, HSE+SOC was adopted to better evaluate the bandgap by considering the relativistic effect of Bi.

**
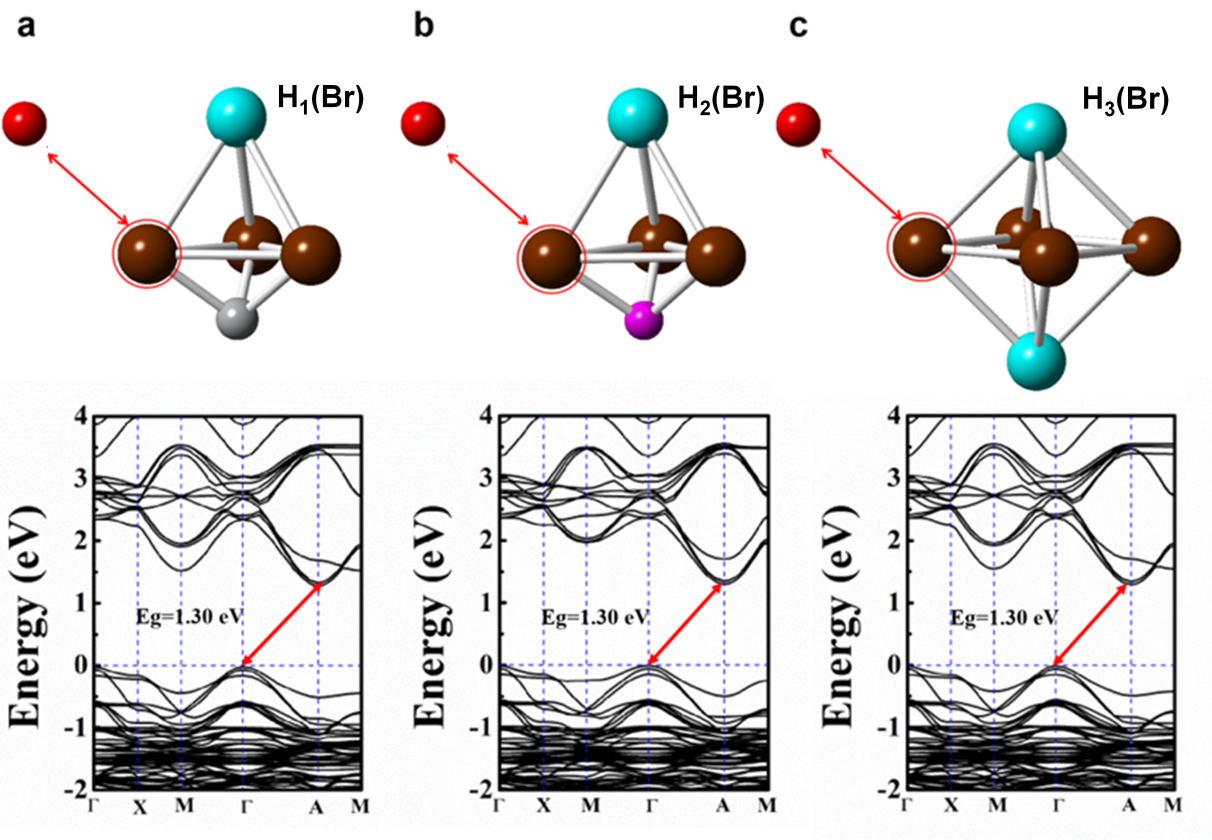
**

**Supplementary Figure 16. The schematic crystal structures and PBE band structures of H^*^ occupying at three different Br vacancies: a** H_1_ polyhedron and H_1_(Br), **b** H_2_ polyhedron and H_2_(Br) and **c** H_3_ polyhedron and H_3_(Br) in Cs_2_AgBiBr_6_. Cs, Ag, Bi, Br and H atoms are represented by cyan, light grey, purple, brown and red dots, respectively. The band structures show that the three types of H_n_(Br) (n=1, 2, 3) have PBE-bandgaps of ~1.30 eV, which is equal to the one in host Cs_2_AgBiBr_6_ (Supplementary Fig. 15a). Whereas, experimental observation shows that the bandgap gradually decreased with the increasement of hydrogenation time (Fig. 1d). So, the substitution of H atoms to Br sites are not the main contribution for the optimization of bandgap in hydrogenated Cs_2_AgBiBr_6_.


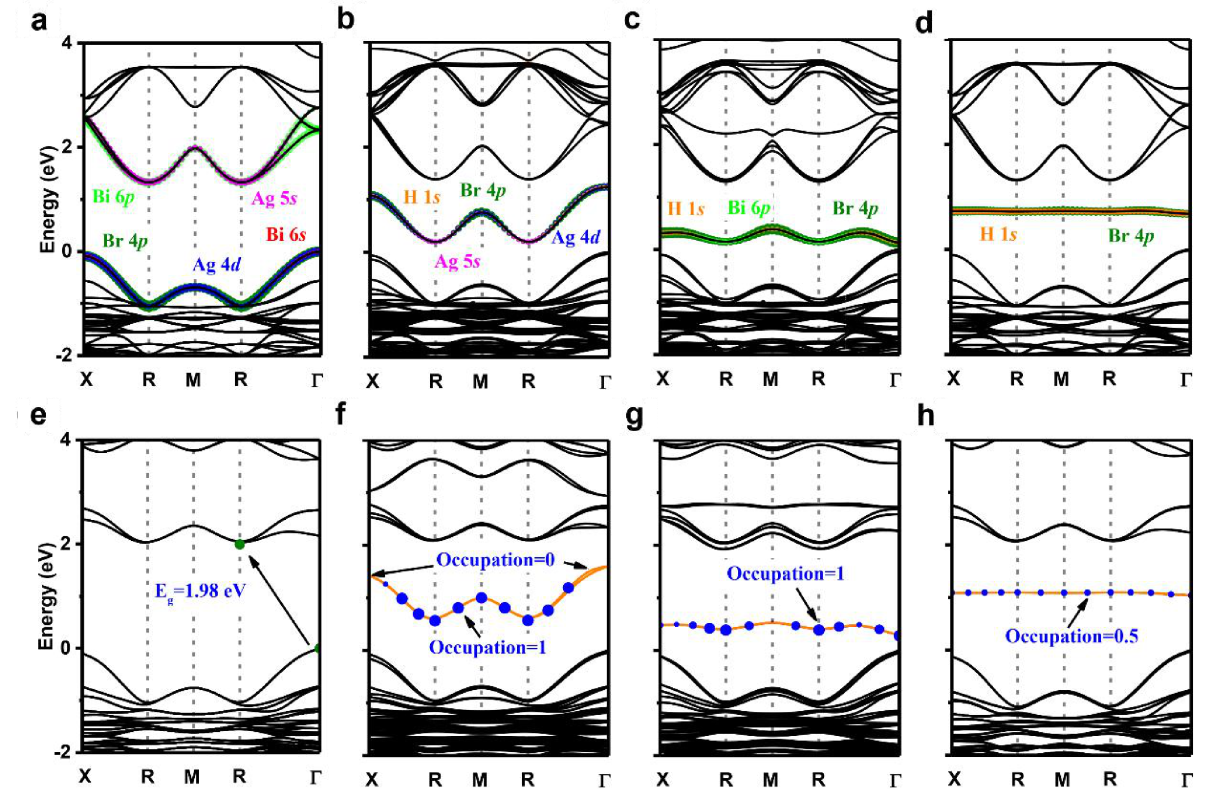


**Supplementary Figure 17. PBE band structures of a** pure Cs_2_AgBiBr_6_ (host) and **b-d** with interstitial H^*^ including: **b** H_1_(in), **(c)** H_2_(in), and **d** H_3_(in). The band edges for host and interbands for interstitial H^*^ are highlighted. Red, light green, blue, pink, dark green, and orange curves correspond to Bi 6*s*, Bi 6*p*, Ag 4*d*, Ag 5*s*, Br 4*p* and H 1*s* orbitals, respectively. **e-h** The HSE+SOC band structures of Cs_2_AgBiBr_6_ (host) and host+interstitial H^*^ containing H_1_(in), H_2_(in), and H_3_(in). The partially occupied inter bands are marked with blue dots, whose size represent the occupation at different k-points along the high symmetry line. The size of occupation=1 is pointed out for reference. The lower conduction band of the host is predominantly antibonding Bi-6*p* and Br-4*p* states, while the upper valence band is the coupling of the Ag-4*d* orbital with the Br-4*p* and Bi 6*s* orbitals. For H_n_(in) induced band structures, they are mainly derived from the bonding states of H-1*s* with nearby cations. In detail, H_1_(in) would form a wide band which is the coupling of H-1*s*, Br-4*p* and Ag-4*d*, while H_2_(in) would introduce a narrow one which is the coupling of H-1*s*, Br-4*p* and Bi-6*p*. However, H_3_(in) form a flat band consisting of H-1*s* and Br-4*p*, which was resulted from the H locating at the center of centrosymmetric octahedron.


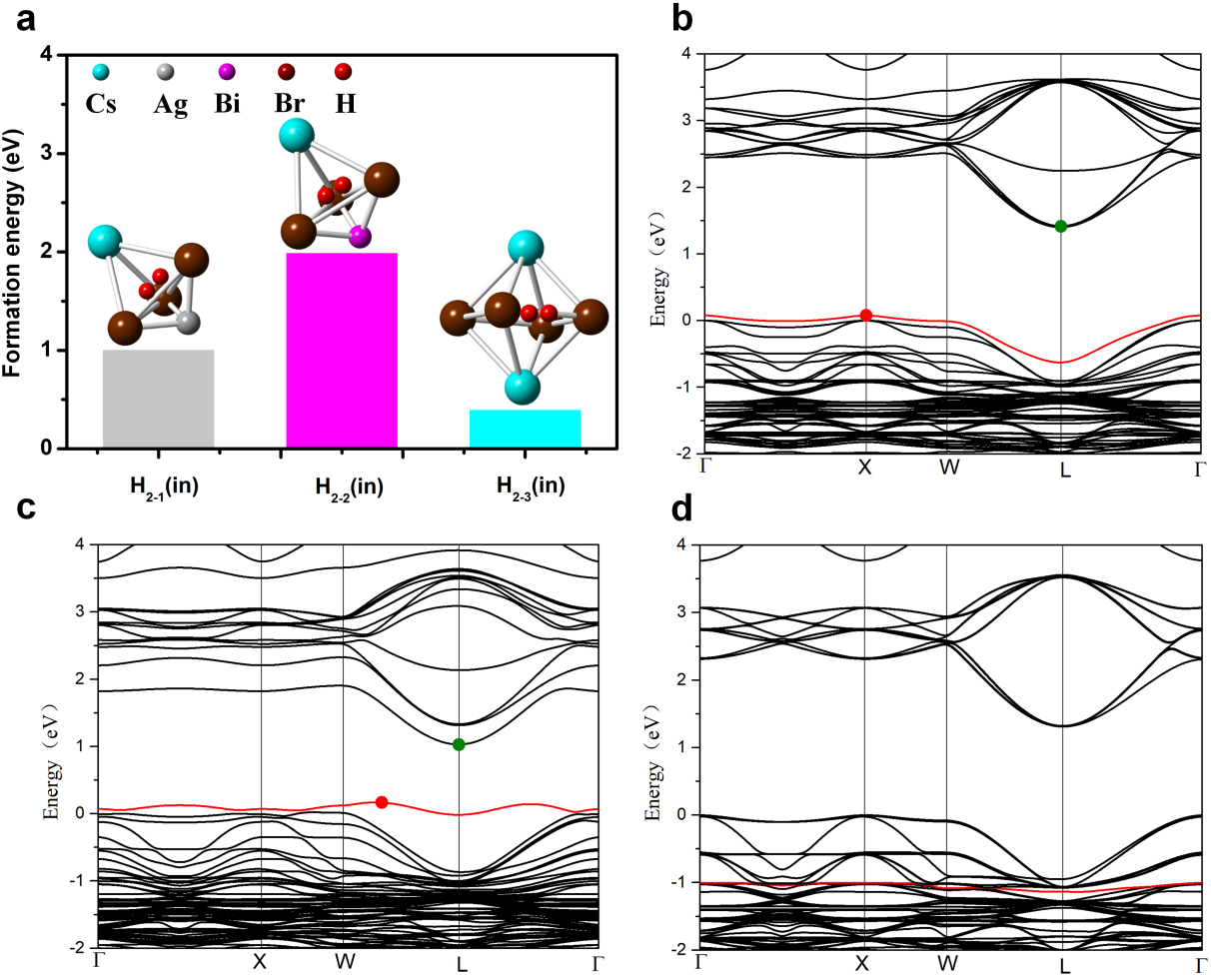


**Supplementary Figure 18. a** The formation energy of hydrogen molecule into the lattice of Cs_2_AgBiBr_6_. There are three position named H_2-1_(in), H_2-2_(in) and H_2-3_(in). Here, H_2_-n(in), where n=1, 2, or 3, presents the H_2_ molecules in the interstitial position of the H_n_ polyhedrons surrounded by Cs-Br-Ag (H_1_), Cs-Br-Bi (H_2_), and Cs-Br-Cs (H_3_), respectively. PBE band structures of Cs_2_AgBiBr_6_ with interstitial H_2_ molecules including: **b** H_2-1_(in), **c** H_2-2_(in) and **d** H_2-3_(in). For the configuration of H_2-1_(in) and H_2-3_(in), both the energy level of CBM and VBM are with almost no change as compared with the ones in host Cs_2_AgBiBr_6_ (Supplementary Fig. 15a), which apparently fit not well with the decreased bandgap in Fig. 1d. While, for the configuration of H_2-2_(in), although the bandgap seems to be reduced. However, with the reduction of CBM, the new band only improves the energy level of valence state, which is against with the experimental values in Fig. 2e.





**Supplementary Figure 19.** The trap density profiles in Cs_2_AgBiBr_6_ layer with different hydrogenation time (0 s, 600 s and 1,200 s), which are detected by using drive-level capacitance profiling (DLCP). The trap density keeps the same level of about 9.0×10^15^ cm^-3^ at 0 s-1,200 s hydrogenation treatment, with almost the same order of magnitude as compared with the reference 19. It means there is almost no trap state been introduced into Cs_2_AgBiBr_6_ layer during the first 1,200 s hydrogenation.


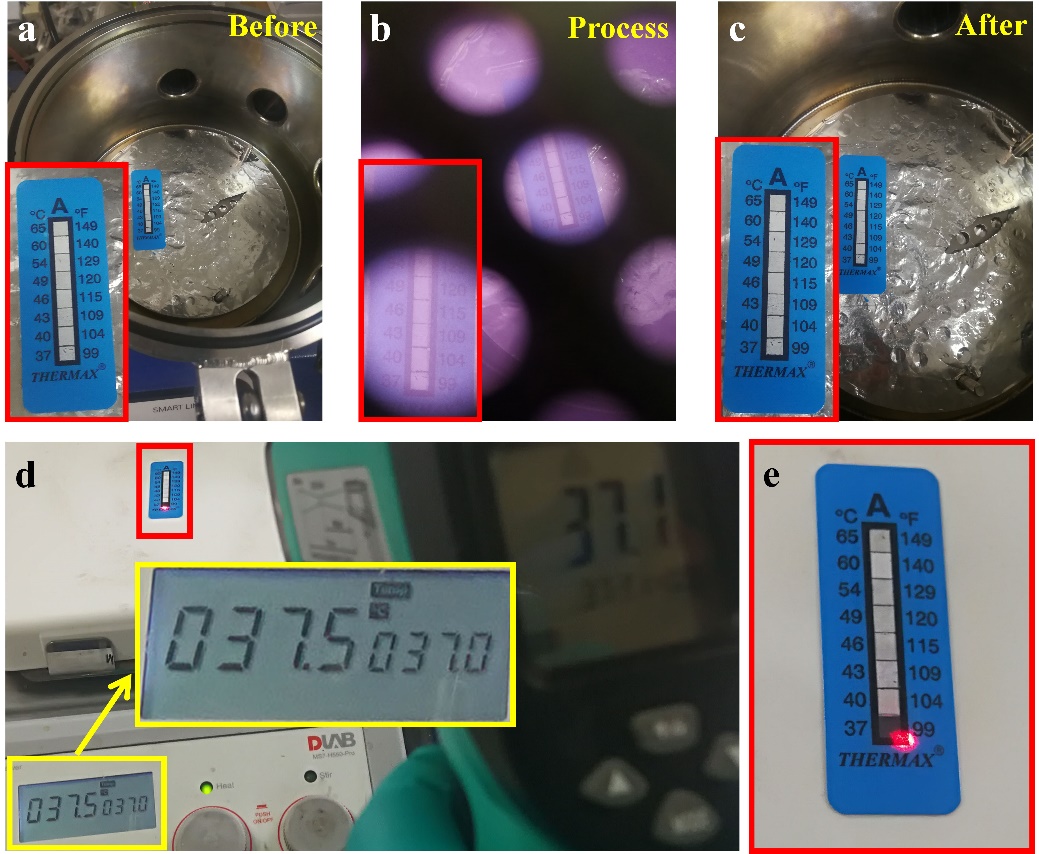


**Supplementary Figure 20.** **a** Before the plasma treatment, **b** Processing the hydrogenation treatment, **c** After 1,200 s hydrogenation (hydrogenation treatment has been completed), Irreversible Surface Temperature Indicating Strips show almost no color change during the whole process. **d** and **e** After that, the Irreversible Surface Temperature Indicating Strips was heating up to 37 ^o^C and the temperature was also calibrated by the infrared thermometer. Optical images show that the Irreversible Surface Temperature Indicating Strips is not affected by the plasma treatment. So, the processing temperature during plasma treatment is below 37 ^o^C.

**Supplementary Table 1.** The carrier lifetime of Cs_2_AgBiBr_6_ perovskite films with different hydrogenation time.

|  | τ_1_ (ns) | Rel_1_ (%) | τ_2_ (ns) | Rel_2_ (%) | τ_3_ (ns) | Rel_3_ (%) | τ (ns) |
| --- | --- | --- | --- | --- | --- | --- | --- |
| Pristine | 1.70 | 35.82 | 6.57 | 25.13 | 46.48 | 39.05 | 18.85 |
| 600 s  Hydrogenation | 2.36 | 29.75 | 10.51 | 22.37 | 61.57 | 47.88 | 30.67 |
| 1200 s Hydrogenation | 1.91 | 17.98 | 8.45 | 17.04 | 55.79 | 64.98 | 41.86 |
| 1800 s Hydrogenation | 1.46 | 15.98 | 6.26 | 29.32 | 50.41 | 54.70 | 28.61 |
| 2400 s Hydrogenation | 1.45 | 48.92 | 6.46 | 22.63 | 46.14 | 28.44 | 13.58 |

**Supplementary Table 2.** Summary for the diffusion coefficient of H^*^ in different materials.

| Sample | X70  pipeline  steel WM | Pd | Co_0.2_Cr_0.2_  Fe_0.2_Mn_0.2_  Ni_0.2_ | 100Cr6  steel | LaNi_4.25_  Al_0.75_ | (Y_2_O_3_)_8_  (ZrO_2_)_92_ | Titanium  oxide | CuInSe_2_ |
| --- | --- | --- | --- | --- | --- | --- | --- | --- |
| D (cm^2^/s) | 1.07×10^-5^ | 2.6×10^-7^ | 1.8×10^-7^ | 4.9×10^-8^ | 2.97×10^-11^ | 2×10^-13^ | 9.5×10^-15^ | 4.9×10^-15^ |
| Ref | 45 | 46 | 47 | 48 | 49 | 50 | 51 | 52 |

**Supplementary Table 3.** The simulated$\frac{C}{C_{s}}$ values as a change of hydrogenation time and H^*^ diffusion coefficient in the 140 nm thick hydrogenated Cs_2_AgBiBr_6_ perovskite films (extracting from the Supplementary Fig. 4).

| Hydrogenation  time (s)  C/C_s_  D (cm^2^/s) | 600 s | 1200 s | 1800 s | 2400 s | 3000 s | Description |
| --- | --- | --- | --- | --- | --- | --- |
|  | 0.205 | 0.405 | 0.834 | 0.963 | 1.000 | Experimental C'/ C_s_' value |
| 1×10^-15^ cm^2^/s | 0.000 | 0.000 | 0.000 | 0.000 | 0.000 | Impossible |
| 1×10^-14^ cm^2^/s | 0.000 | 0.004 | 0.020 | 0.046 | 0.074 | Possible |
| 5×10^-14^ cm^2^/s | 0.074 | 0.200 | 0.298 | 0.369 | 0.419 | Possible |
| 1×10^-13^ cm^2^/s | 0.203 | 0.366 | 0.460 | 0.522 | 0.571 | Possible |
| 2×10^-13^ cm^2^/s | 0.368 | 0.521 | 0.603 | 0.653 | 0.685 | Impossible |
| 1×10^-12^ cm^2^/s | 0.685 | 0.775 | 0.817 | 0.838 | 0.858 | Impossible |
| 1×10^-10^ cm^2^/s | 0.970 | 0.977 | 0.982 | 0.984 | 0.985 | Impossible |
| 1×10^-5^ cm^2^/s | 1.000 | 1.000 | 1.000 | 1.000 | 1.000 | Impossible |

Here *C_s_* is the concentration of hydrogen at the surface of perovskite film, and *C* is the atomic hydrogen concentration at the film depth of 140 nm. Assuming the blackness values of the front and back side images (in Fig. 2a) are respectively proportional to the average H^*^ concentration near the top and bottom surface of hydrogenated Cs_2_AgBiBr_6_ film, as shown by *C_s_ˊ* and *Cˊ*; then the blackness ratio of the back and front side images can be used as the reference ratio of $\frac{C'}{C_{s}'}$ for judging the rationality of the calculated $\frac{C}{C_{s}}$values.

**Supplementary Table 4.** Summary for the evolution of cubic crystal lattice parameter of Cs_2_AgBiBr_6_ after doping different concentration of hydrogen. The hydrogen concentration indicates the number of hydrogen atoms in the total number of atoms in hydrogenation Cs_2_AgBiBr_6_ lattice during first-principle calculation.

| Hydrogen Concentration | Doping Type | Lattice Constant (Å) | |
| --- | --- | --- | --- |
|  |  | Calculated | Average |
| 0 | Pristine | 11.276 | 11.276 |
| 1/320 | H_1_-(i) | 11.296 | 11.290 |
|  | H_2_-(i) | 11.291 |  |
|  | H_3_-(i) | 11.283 |  |
| 1/160 | H_1_-(i) | 11.301 | 11.299 |
|  | H_2_-(i) | 11.311 |  |
|  | H_3_-(i) | 11.284 |  |
| 1/40 | H_1_-(i) | 11.346 | 11.329 |
|  | H_2_-(i) | 11.354 |  |
|  | H_3_-(i) | 11.287 |  |

**Supplementary Table 5.** Langmuir probe detection on the average electron temperature in plasma chamber under different working condition.

| Power/W | Working Pressure/Pa | Position | Average Electron  Temperature/eV |
| --- | --- | --- | --- |
| 20 | 1.4 | Center | 9.0 |
| 30 | 1.4 | Center | 9.1 |
| 40 | 1.4 | Center | 9.3 |
| 40 | 1.4 | Side | 9.3 |

**Supplementary Table 6.** Summary for bond dissociation energy in Cs_2_AgBiBr_6_.

|  | Ag-Br | Bi-Br | Cs-Br |
| --- | --- | --- | --- |
| Bond Dissociation Energy (eV) | 2.91 | 2.49 | 4.03 |

**References**

1. Xiao, Z. W., Meng, W. W., Wang, J. B. & Yan, Y. F. Thermodynamic stability and defect chemistry of bismuth‐based lead‐free double perovskites. *Chem. Sus. Chem.* **9**, 2628-2633 (2016).

2. Feng, Y. H., Ke, X. X. & Sui, M. L. Effect of electron irradiation on inorganic double perovskite solar cell material Cs_2_AgBiBr_6_. *J. Chin. Electr. Microsc. Soc*. **39**, 1-8 (2020).
