# Supplementary material for: Hydrogenated Cs2AgBiBr6 for significantly improved efficiency of lead-free inorganic double perovskite solar cell
Source: Nat Commun. 2022 Jun 13;13:3397. doi: 10.1038/s41467-022-31016-w (PMC9192601; doi:10.1038/s41467-022-31016-w)
Supplement: Supplementary file 3 — Solar Cells Reporting Summary [file 41467_2022_31016_MOESM3_ESM.pdf]

nature research

Corresponding author(s): Manling Sui

# Solar Cells Reporting Summary

Nature Research wishes to improve the reproducibility of the work that we publish. This form is intended for publication with all accepted papers reporting the characterization of photovoltaic devices and provides structure for consistency and transparency in reporting. Some list items might not apply to an individual manuscript, but all fields must be completed for clarity.

For further information on Nature Research policies, including our data availability policy, see Authors & Referees.

## Experimental design

**Please check: are the following details reported in the manuscript?**

1. Dimensions

| Item | Yes | No | Details |
| --- | --- | --- | --- |
| Area of the tested solar cells | ☒ | ☐ | The area of the tested solar cells is 0.04 cm2(Method, Characterization of solar cells and films) |
| Method used to determine the device area | ☒ | ☐ | The active area was determined by the shading mask which certified by National Institute of Metrology, China (Method, Characterization of solar cells and films) |

2. Current-voltage characterization

| Item | Yes | No | Details |
| --- | --- | --- | --- |
| Current density-voltage (J-V) plots in both forward and backward direction | ☒ | ☐ | Figure 3b |
| Voltage scan conditions<br>*For instance: scan direction, speed, dwell times* | ☒ | ☐ | JV curves were measured by reverse scan (1.2 V to -0.2 V) and forward scan (-0.2 V to 1.2 V) with a scanning rate of 200 mV/s (voltage step of 20 mV and delay time of 100 ms) (Method, Characterization of solar cells and films) |
| Test environment<br>*For instance: characterization temperature, in air or in glove box* | ☒ | ☐ | Performance measurements were carried in air ambient environment (Main text) |
| Protocol for preconditioning of the device before its characterization | ☐ | ☒ | No preconditioning was used |
| Stability of the J-V characteristic<br>*Verified with time evolution of the maximum power point or with the photocurrent at maximum power point; see ref. 7 for details.* | ☒ | ☐ | Maximum power point measurements were conducted (Figure 3c and Supplementary Figure 8) |

3. Hysteresis or any other unusual behaviour

| Item | Yes | No | Details |
| --- | --- | --- | --- |
| Description of the unusual behaviour observed during the characterization | ☒ | ☐ | The hydrognated Cs2AgBiBr6 solar cell showed moderate hysteresis |
| Related experimental data | ☒ | ☐ | Figure 3b |

4. Efficiency

| Item | Yes | No | Details |
| --- | --- | --- | --- |
| External quantum efficiency (EQE) or incident photons to current efficiency (IPCE) | ☒ | ☐ | Figure 3e |
| A comparison between the integrated response under the standard reference spectrum and the response measure under the simulator | ☒ | ☐ | The integrated Jsc from EQE spectra is consistent with the Jsc from JV measurements (Figure 3a and Figure 3e) |
| For tandem solar cells, the bias illumination and bias voltage used for each subcell | ☐ | ☒ | No tandem cells reported in this manuscript |

5. Calibration

| Item | Yes | No | Details |
| --- | --- | --- | --- |
| Light source and reference cell or sensor used for the characterization | ☒ | ☐ | We used Xenon arc lamp solar simulator and silicon reference solar cell |
| Confirmation that the reference cell was calibrated and certified | ☒ | ☐ | The light source is celibrated using standard silicon solar cells (Isc=130.5 mA), the reference silicon solar cell is certified by National Institute of Metrology |

Calculation of spectral mismatch between the reference cell and the devices under test

- [ ] Yes
- [x] No

The light spectrum used for measurements can match with the reference silicon cell, and we did not calculate the spectral mismatch between the reference cell and the tested devices

6. Mask/aperture

Size of the mask/aperture used during testing

- [x] Yes
- [ ] No

0.04 cm2 (Method, Characterization of solar cells and films)

Variation of the measured short-circuit current density with the mask/aperture area

- [ ] Yes
- [x] No

We did not measure the perovskite solar cells with different mask areas

7. Performance certification

Identity of the independent certification laboratory that confirmed the photovoltaic performance

- [ ] Yes
- [x] No

We did not certificated the photovoltaic performance

A copy of any certificate(s)
*Provide in Supplementary Information*

- [ ] Yes
- [x] No

We did not certificated the photovoltaic performance

8. Statistics

Number of solar cells tested

- [x] Yes
- [ ] No

At least 50 devices were tested (Figure 3d and Supplementary Figure 9)

Statistical analysis of the device performance

- [x] Yes
- [ ] No

Figure 3d and Supplementary Figure 9

9. Long-term stability analysis

Type of analysis, bias conditions and environmental conditions
*For instance: illumination type, temperature, atmosphere humidity, encapsulation method, preconditioning temperature*

- [x] Yes
- [ ] No

Long-term stability including light illumination (20 oC under AM 1.5 G simulated solar illumination at 100 mW cm-2 in N2 glovebox), 85 oC (20 oC dark in N2 golvebox), 85 oC plus light illumination (85 oC under AM 1.5 G simulated solar illumination at 100 mW cm-2 in N2 glovebox) and 85% humidity at 85 oC stability (dark) were tested (Figure 3f).
